# Are algorithmically controlled gig workers deeply burned out? An empirical study on employee work engagement

**DOI:** 10.1186/s40359-023-01402-0

**Published:** 2023-10-24

**Authors:** Jiao Jiao Lang, Li Feng Yang, Chen Cheng, Xiang Yang Cheng, Fei Yu Chen

**Affiliations:** 1 Endicott College, Woosong University, 17-2, Jayang- dong, Dong-gu, Daejeon City, South Korea; 2 School of Economics, Fuyang Normal University, No. 100, Qinghe West Rd, Ying Zhou District, Fuyang City, An Hui Province China; 3 School of Business, Fuyang Normal University, No. 100, Qinghe West Rd, Ying Zhou District, Fuyang City, An Hui Province China; 4 School of Economics and Management, Fei Yu Chen, China University of Mining and Technology, No. 1 University Road, Xuzhou City, Jiangsu Province China

**Keywords:** Gig economy, Algorithmic technology, Flow experience, Employee work engagement

## Abstract

**Background:**

With the emergence of the gig economy as a new economic form, the influence of algorithmic technology control on gig workers’ perceptions and engagement has become a topic of academic concern. This study explores the emotional impact of perceived algorithmic control on gig workers and how it affects their work engagement.

**Methods:**

This study takes gig workers as the research object to build a structural equation model. Based on the background of gig economy and the Job Demands-Resources model, this paper constructs a mechanism model of the influence of perceived algorithmic control on the work engagement of gig workers. The research data in this paper are collected by questionnaire, and the research hypothesis is tested by the SEM structural model.

**Results:**

The gig workers in this study believed that perceived algorithmic control positively affects employee work engagement. In addition, burnout was positively correlated with employee work engagement. Burnout played a partial mediating role in the relationship between perceived algorithmic control and employee work engagement. And flow experience played a moderating role through the indirect effect of burnout on employees’ work engagement.

**Conclusion:**

Perceived algorithmic control causes burnout among gig workers, but strong algorithmic technology support provides them with rich work resources that can help them meet their work needs. That is, the gig workers may still demonstrate a high level of work engagement even if they experience burnout symptoms.

## Introduction

Employee work engagement refers to the favourable allocation of personal resources (for example, energy and emotions) to tasks related to job roles [1, 2]. Its most important feature is that employees are attracted by the work they are engaged in and make continuous efforts for it [3]. Employee job engagement plays a crucial role in transforming human resources into situational performance, organizational performance, and sustained competitive advantage [4–6], promoting organisations’ continuous development [7]. However, according to the research, global employee work engagement in different industries remains at only around 15% [8]. In the current era of the COVID-19 pandemic, anxiety surrounding health threats and the isolation of working remotely online have caused employee work engagement to fall to even lower levels [9, 10], and finding ways to motivate employees to work has become a hot topic in the research field [11]. Many studies have shown that personality traits, situational motivation, job centrality, job resources, and organizational climate significantly impact employee work engagement [12, 13]. Dollard et al. (2010) further explained that organizational capacity and climate affect employee work engagement by influencing work resources and work demands [14], which in turn affects employee work engagement [15]. In the changing digital age, new organizational models are evolving and iterating at an accelerated pace, and technological systems and platforms are creating new work contexts that impact social systems while digital organizational control methods are being enhanced to improve employee work engagement [16].

The algorithmic technology-based gig economy (also known as the digital platform economy) [17]is rapidly changing the way organizations are controlled [18], whereby algorithmic technology assumes management functions—breaking spatial constraints and facilitating the growth of the gig economy [19, 20]. As an essential player in the gig economy, gig workers are people who solve complex problems or perform trivial daily tasks online using online intelligent platforms as a medium [21, 22]. Unlike traditional forms of organization, platform companies use algorithmic systems to plan work assignments, assess constraints on gig workers, and set salaries [23], which seems to fit perfectly with the advantages of digital technology. However, in the process of human-computer interaction and cooperation, algorithmic technology pushes information, the deviation correction process, and feedback results for gig workers in real-time with its mighty computing power [24], helping them to meet their work needs. Gig workers gradually find themselves in interpersonal isolation—in a social vacuum, separated from traditional organizational forms and dependent on algorithmic platforms [25].

In addition, digital platform companies use algorithmic technologies to rate, count, and track employees and even control their job opportunities [20], and gig workers appear to be becoming “puppets” of algorithmic control [26]. The increasing refinement of algorithmic technology has led to increasing job demands and workloads [27], and the comprehensive coverage, immediate feedback, and high-frequency interactions of algorithmic technology have overwhelmed gig workers [28]. However, breaking away from the control of the algorithmic system and returning to the freelance gig work environment can contribute to a loss of meaning and group affiliation, generating anxiety and confusion about one’s identity [29]. With limited initiative and increasing control, gig workers are gradually becoming resistant to digital methods of organizational control [30]. Platform work means it is even more difficult for gig workers to stay highly engaged for long periods, and excessive stress causes both physical and mental exhaustion [31–33], leading to the gradual onset of burnout [34]. Highly reinforced algorithmic control causes gig workers to experience negative emotions such as self-denial and anxiety for long periods, which diminishes their sense of self-efficacy, and serious burnout can result [35, 36]. In gig workers, this constant state of being “monitored” and “on-call” triggers bad moods (burnout) and warrants further attention and consideration by research [37, 38].

Researchers have traditionally regarded burnout and work engagement as distinct psychological states within the context of organizational activities [39, 3]. Burnout was first put forward by psychiatrist Freudenberger in 1974, which was used to describe the cognitive and emotional response of nurses to emotional and interpersonal pressure for a long time [40]. Does burnout among gig workers have an impact on their work engagement in the new organizational environment? In previous studies, scholars primarily focused on the influence of personal or environmental factors on employee burnout [41]. However, little has been said about whether digital technology controls itself and triggers negative emotions among employees. The attitude and behavior of gig workers will be fundamentally influenced or shaped by how they feel, recognize, and evaluate algorithmic control in the process of human-computer interaction. Different gig workers have different perceptions and understandings about the practice of algorithmic control and will make different responses accordingly [42]. In the wake of the COVID-19 pandemic, control over data algorithms intensified. Further research is needed to examine the point of whether burnout is triggered during this algorithmic environment and whether the level of work engagement among employees decreases as a result [43]. Cole et al. employed meta-analysis techniques to demonstrate a high correlation between burnout and work engagement [44]. However, the research community is divided regarding its relevance. Fiksenbaum et al. argued that intensive labor control increases employees’ emotional exhaustion risk, adversely affecting their attitudes and behaviors [45, 46]. However, through Latent Profile Analysis (LPA) and configuration frequency analysis, Moeller et al. [47] concluded that some employees have high burnout and high work engagement at the same time. From this point of view, it is of great significance and the research goal of this paper to discuss whether perceived algorithmic control will cause the burnout of gig workers and what impact it will have on their work engagement in the process of gig economic development.

From the perspective of the Job Demands-Resources model [48], this study aimed to investigate adverse emotional responses (burnout) among gig workers in response to perceived algorithmic control [49, 50], to explore whether other relationships between burnout and employee work engagement exist. This paper argues that when gig workers perceive algorithmic control of online labor platforms, a negative emotional response is elicited (burnout). At this time, the individual’s psychology and body are expanded to the extreme, and the flow experience of gig workers is stimulated and impacts their emotions, which in turn will impact employees’ work engagement [51]. The gig workers’ flow experience influences this response and can impact employee work engagement. In the context of the global implications of the COVID-19 pandemic, people are crying out for “involution”, but is it really “lying flat”? The main contributions of this paper are as follows: firstly, the positive relationship between perceived algorithmic control and burnout is verified through empirical research; more importantly, this study verifies the adaptive behavior of gig workers, that is, gig workers may still show high work engagement when they are deeply burnout. Given this situation at the time of writing, the current research has more theoretical and practical significance.

## Literature review and hypotheses

### Perceived algorithmic control

French thinker Gilles Deleuze states that social control is through continuous control and instant information dissemination. So, is this the case with algorithmic control in the digital age [52]? Under the new organizational form, more decision-making freedom and task autonomy are the autonomy that employees hope to obtain in organizational activities [53, 54], particularly considering the Job Demands-Resources(JD-R)model, which regards employee autonomy as a vital work resource that promotes work engagement [48, 55]. However, employee autonomy will be accompanied by personal preferences, employee characteristics, and other personalized problems, which means that organizations are confronted with the dilemma of uncertainty management [56]. Employee autonomy without supervision is more likely to cause a deviation from management objectives [57], so organizations must constantly balance the relationship between organizational control and employee autonomy to ensure and improve the efficiency of the enterprise’s management. Algorithmic technological advances have broadened the scope and created greater precision in organizational management [58], causing a gradual breakdown in the equilibrium between employee autonomy and organizational management [59]. However, employee autonomy can enhance employee self-efficacy and reduce work stress, impacting company performance [60]. Algorithmically supported organizational supervision and control cause employees to feel overloaded with stress and negative emotions like tension and worry [61]. The impact of advances in information technology on organizational management capabilities and business practices varies [62]. In the gig industry, which is based on algorithmic technology, the extent to which the balance between employee autonomy and organizational management is broken becomes more significant [63].

Gig workers rely on algorithmic technology to provide resources such as information, services, salary assessments, and even job opportunities [64, 65]. Therefore, algorithmic techniques are more evident in controlling gig workers [63]. Online intelligent platforms design platform rules and processes based on Artificial Intelligence (AI) algorithms [65], which automatically output control functions and optimize control levels—termed algorithmic control [49, 66]. Algorithmic control replaces manual supervision for gig workers in real-time to ensure work engagement [63], triggering overcommitment of work time and emotional exhaustion [45, 46]. The continuous advances in algorithmic technology also drive changes in organizational control capabilities and approaches [16, 67]. Algorithms run with the help of computerized deep algorithmic computations [68] that precisely set steps and operating rules, and organizations can solve complex problems such as autonomous management with the help of intelligent algorithms [58]. Online intelligent platforms use algorithmic technology to innovate business models, exploit competitive advantages, fully exploit the value of human capital in the gig economy market [69], and enhance platform management capabilities [70]. Scholars argue that algorithmic management, with the advantage of intelligent algorithms, assists decision-makers in making scientifically rational decisions [71], achieves rapid and accurate matching of labor and demand [72], and dramatically improves the efficiency of labor use [73]. Online intelligence platforms build a digital virtual space manipulated by algorithmic technologies [74] that control the entire workflow of gig workers [75]. Although the division of labor in today’s fractional economy differs from that of employees in the traditional sense (Cappelli et al.,2013) [76], the gig workers under the algorithmic control of the digital economy may experience a higher degree of organizational control [77]. Perceived control is a belief that the outcome depends on the individual action itself and is unrelated to the external environment [78]. Gig workers use their feelings and perceptions to take in information under the labor platform’s control and translate this idea into work actions, such as the need to deliver items to a specified location within a certain time or lose money. This process shows that gig workers accept the level of algorithmic control and understand and internalize the service information as well as the reward and punishment measures provided by the online labor platform [79]. These internalized value judgments will unconsciously affect their recognition and commitment to the work.

### Perceived algorithmic control and employee work engagement

Algorithmic control refers to the process in which the online labor platform uses algorithmic technology to monitor the behavior of gig workers and ensure that their behavior is consistent with the requirements of digital platform enterprises [80]. However, the dynamic interaction between different individuals and the environment will generate different cognition and evaluation. Individuals may have different perceptions of the same event or situation [81]. The gig worker’s perception of algorithmic control describes their perception and cognition of algorithmic technical control. When there is a deviation in perception, the inconsistency between organizational and personal expectations will lead to different work engagements of individuals [82].

Employees’ vitality, dedication, and dedication in their work are the remarkable characteristics of high employee job engagement [3], improving employees’ customer service level and affecting organizational service performance [83]. In the service situation of platform enterprises, gig workers with a higher perception of algorithmic control have higher self-satisfaction and self-efficacy [84] and are more likely to exhibit supportive organizational behaviors [85]. Although the online platform controls gig workers through big data intelligence algorithms, the algorithm is inherently lacking in sympathy and empathy [42, 86], it is undeniable that algorithmic control affects employees’ work engagement by controlling factors such as situational motivation and work resources [13]. As far as the new organizational model algorithmic technology is concerned, reducing the cost of organizational salary management and improving the effectiveness of the reward and punishment system is particularly important to platform companies. However, this digital control method also means that gig workers lose the right to speak about job assignments and performance evaluation [87]. Confronted with a digital organizational environment that is subject to a rising level of control, the adaptive behavior of gig workers is constantly being stimulated [88]. In other words, given the “comprehensive monitoring” system of the perception algorithm, gig workers may have stronger self-presentation motivation and more persistent behavior [86]. The powerful computing power of algorithmic technology also requires massive work resources and information. From the perspective of work demand-resource theory, employees are more dedicated to their work and more productive when there is a balance between their work needs and work resources [89]. A review of recent studies reveals that scholars largely believe that algorithmic control can help platform companies to organize and manage their practices and avoid risks and uncertainty in human resource management, platform operations, and employee service quality brought about by flexible employment [58, 75]. Perceived control is associated with psychological empowerment and enhances gig workers’ perceptions of their self-worth, which is more pronounced in tasks related to job roles [89, 90]. Therefore, this study proposes that, about online labor platforms, gig workers who have a stronger perception of algorithmic control exhibit higher levels of self-worth and adaptability and thus demonstrate higher levels of employee work engagement. In summary, we put forward the following hypothesis:

#### Hypothesis 1

Perceived algorithmic control is positively correlated with employee work engagement.

### The mediating role of burnout

There is a need to conduct further research to examine whether controlling highly intelligent algorithm technology on gig workers will cause negative emotions, such as burnout and insecurity [49, 50]. Scholars have come up with contradictory research conclusions on the above issues.

Some studies have pointed out that algorithmic control can encourage gig workers to have a positive emotional experience and promote behaviors such as excitement and fairness [91]. However, many studies have demonstrated that, among gig workers, algorithmic technology means that the labor process is subjected to comprehensive monitoring, due to the implementation of a precise and meticulous management system [92]. Continuous long-term work and greater labor intensity hurt physical health and contribute to the development of mental health problems. These factors can significantly diminish job satisfaction and the well-being of gig workers, and trigger emotional exhaustion [45, 46]. Increased labor intensity means that the body is deprived of sufficient rest, and the continuous consumption and sub-optimal physical health can cause gig workers to have low energy levels, which negatively affects their mood [93]. Many gig workers are often online 24/7 due to the uncertainty of their work assignments. A work-family balance is difficult to attain for gig workers who find themselves in a difficult financial situation or who have many family responsibilities [50]. Wood et al. also believed that the continuous control of algorithmic systems violates the flexible and autonomous working state advocated by online intelligence platforms. In addition, gig workers have voiced their concerns about the impact of algorithmic evaluations on their reputation and income, which undoubtedly intensifies feelings of insecurity and burnout [49].

For gig workers, their jobs are characterized by high risk and uncertainty. Many can feel overwhelmed by strictly controlled environments managed by platforms through algorithms, which causes them to feel confused about their identity, which contributes to burnout [75]. Online labor platforms rely on algorithmic technical means to strictly manage and control gig worker labor as a data storage asset [26, 91]. However, gig workers can only obtain compensation by relying on the information provided by the platform algorithm and by completing the work according to the workflow instructions, essentially the online platform’s use of algorithmic technology to dominate the control of labor power [91]. With this level of control, it is reasonable to argue that algorithmic control triggers burnout among gig workers.

The term “burnout” was first used to describe the mental state in which people who volunteered to work for aid organizations in New York experienced a gradual decline in their mood and energy levels [94]. The premise of this notion was based on the belief that the volunteers were described as energetic at work before the manifestation of these symptoms [95]. Based on the JD-R model, it is assumed that job burnout is caused by an imbalance between job requirements and job resources [48]. Combined with the JD-R model extension model [96], employees will experience potential psychological processes under work pressure and motivation. The psychological process means that high job demands deplete employees’ energy levels and physical strength, which may cause health problems or job burnout. In conclusion, this study argues that when gig workers are fully aware of the algorithm’s control over them, they will experience burnout.

#### Hypothesis 2

Perceived algorithmic control is positively correlated with burnout.

According to relevant studies, burnout refers to a slow process that is characterized by chronic emotions and can be understood as a response to interpersonal stressors, manifesting in the form of a gradual decline of energy and enthusiasm at work [97, 98]. In organizational activities, employees use their cognition and emotion to interpret their job roles and responsibilities [99]. Studies on burnout and employee job engagement have attracted increasingly more attention from scholars [40, 100].

Early studies, measured mainly by the Maslach Burnout Scale, concluded that work engagement is associated with positive emotions [4] and that burnout is antithetical to employee engagement [101]. When employees feel physically burned out, their energy levels and sense of efficacy are reduced [35]. As research continues, burnout—a negative emotion that is positively associated with employee work engagement—has attracted the attention of researchers [102]. Xie (2021) and others have demonstrated that it is the perception of being in a “panoramic prison” and under constant observation that may stimulate a stronger motivation for self-presentation and possibly cause more persistent behavior [103]. Shevchuk and Liu et al. similarly noted that since gig workers rely heavily on the technical support provided by algorithms, they are subjectively willing to continuously perform job reinforcement under algorithmic control [50, 63]. From this point of view, gig workers are likely to show high levels of work engagement even if they experience burnout arising from their perceptions of algorithmic control, which is consistent with the JD-R model. According to Demerouti’s JD-R model and Schaufeli’s JD-R extension model, job resources are potentially motivating, since they promote work engagement, reduce interpersonal apathy, and stimulate high performance [104]. Moreover, the intrinsic motivational nature of work resources can promote employee growth and advancement [48, 96]. Strong algorithmic technology support provides gig workers with rich work resources, which thus helps to ensure that their gig jobs meet their work demands. Moreover, these workers may still demonstrate high levels of work engagement even when they feel burned out. Moeller also argues that individuals can be exhausted and highly engaged [47]. It is common to “face difficulties” at work [100], which means that they need to devote more time and energy to solving even complex tasks [105]. In summary, the following hypothesis is proposed:

#### Hypothesis 3

Burnout is positively correlated with employee work engagement.

#### Hypothesis 4

Burnout mediates the relationship between perceived algorithm control and employee work engagement.

### The moderating role of flow experience

At a time when artificial intelligence (AI) is advancing globally, psychological perception is attracting more and more attention in the field of human-technology relations [106]. From the perspective of human-machine interactions, individual responses to machine control originate to some extent from the level of psychological and mental state [107]. Csikszentmihalyi thinks that Flow Experience describes the mental state in which an individual is completely absorbed in the current activities and automatically filters out the irrelevant consciousness, which is an optimal mental state [108]. Some scholars also argue that flow experience seems to be a state between stress and relaxation, and it is at a moderate level of excitement [109], which makes people feel distorted in time, ignore the surrounding environment, concentrate on the current activities and get happiness from them, and can stimulate individuals’ fantastic creativity and work engagement [110].

Flow experience is an active behavior. Employees in this state are in the best psychological and physical state and tend to think that the feeling of doing it is the best reward [111]. People are more focused on their activities and have less self-awareness when they are in a “flowing state”, and they will feel that they are controlling the environment [112]. It is a chronic pressure for gig workers in the digital age to feel algorithm control and feel burnout [113]. Studies have shown that flow experience exists in stress-related situations [108], which are regarded as challenges [114]. After the flow experience is stimulated, employees are more focused on their work, do not need external rewards, and have the consciousness of problem-solving and innovation [115]. Employees will have a sense of satisfaction and pleasure, which will lead to continuous enthusiasm for activities [51]. Especially when individuals encounter difficulties at work, this state of flow will prompt employees to regard overcoming stress as a challenging task, which will help to stimulate employees’ enthusiasm for work and deal with various problems flexibly [116]. Flow experience is related to some positive results, such as work engagement and creativity, happiness, health, etc. [117, 118]. Hoffman & Novak conceptualizes flow experience as a positive cognitive state, which requires four core elements: high-level love, clear sense of purpose, high-level challenge and skill balance, and timely feedback [119]. Work engagement is also a positive state. Schaufeli et al. (2002) think that work engagement refers to a positive and complete emotional and cognitive state related to work, which can keep concentration without fatigue, has the characteristics of persistence and dispersion, and is characterized by vitality, dedication, and concentration, indicating that a person has enough toughness and energy and is willing to make continuous efforts in his work [3]. Some scholars have studied the relationship between flow experience and employees’ work engagement, and it is found that the flow experience process is conducive to forming work engagement [120, 121]. Akiva et al. (2013) also showed that flow experience can improve employees’ job engagement [122].

Based on previous studies, this study believes that flow experience can weaken the burnout of gig workers and promote the work engagement of employees. Furthermore, this paper quotes Csikszentmihalyi’s view that flow experience emphasizes the overall experience people feel when they are fully involved in actions, so this study discusses flow experience as a single dimension rather than multiple dimensions. Accordingly, this paper proposes the following hypotheses:

#### Hypothesis 5

Flow experience plays a moderating role between burnout and employees’ work engagement. Specifically, gig workers with better flow experience will have higher work engagement.

#### Hypothesis 6

**Fig. 1:**
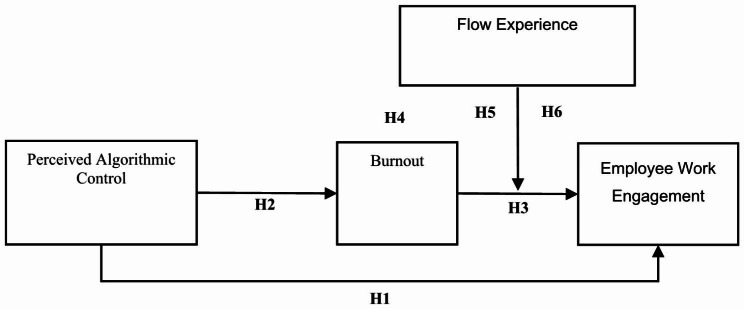
The influence mechanism model of PAC (Perceived Algorithmic Control) on EWE (Employee Work Engagement)

Flow experience has an indirect effect on employee work engagement through burnout and takes the form of a moderated mediation model. In summary, the theoretical model of this study is shown in Fig. 1:

## Research design

### Data collection and design

This paper focuses on several digital platform companies from two provinces in China and mainly collects receipts from people who work through digital media, such as delivery workers from “Ele. Me” and “Meituan” food delivery platform companies and online car-hailing drivers from Didi appeared. The respondents were all adults. The data collection process is legal, and informed consent was obtained from all subjects. To ensure the reliability and pertinence of the data, the prior sample was recruited through the online panel provider WenJuanXing. According to the results of the prior sample, the data quality of samples recruited by online panel companies is comparable to the quality of samples obtained through conventional data sources. In order to reduce concerns about data bias, formal survey data collection adopts two ways:: Firstly, the research group received strong support from the senior managers of enterprises through social resources channels, and emphasized the anonymity and academic research purpose of the questionnaire, which was distributed to the target groups with the cooperation of the human resources department of companies; second, visit the gathering places of the above groups in their spare time to find the target population to fill in.

In this paper, confirmatory factor analysis is used to test the rationality of the measurement model, including the reliability, aggregation validity and discrimination validity of each variable. In addition, according to the research theoretical assumptions, the paper constructs the SEM structural equation model to verify the influence path between variables.

### Variable measuring tool

To ensure the accuracy and reliability of the measurement tools, this study utilizes well-recognized, mature scales with verified reliability and validity through meticulous examination by domestic scholars and testing within Chinese samples. Given that the participants in this study are Chinese, we carefully adapted authoritative foreign English scales to better align with local culture and contexts through a rigorous “translation-back translation” process. By establishing a dedicated translation group, we ensured the accuracy and scientific integrity of the questionnaire items, making the language more congruent with organizational and situational culture in China, thus enhancing cultural applicability and participants’ comprehension and acceptance. All questionnaire items were rated on a five-point scale, ranging from 1 (strongly disagree) to 5 (strongly agree).

Perceived algorithmic control (PAC): We measure PAC using the scale [77], where there are 11 items, such as “The algorithm intelligently assigns my work tasks” “The algorithm locates my geographical position in real time”. The scale has three dimensions, and Cronbach’s α values are 0.810, 0.857 and 0.801 respectively. A confirmatory factor analysis (CFA) was performed and indicated that the scale demonstrated a satisfactory fit with the data: χ2/df = 2.211 < 3, GFI = 0.959, NFI = 949, CFI = 0.971, TLI = 0.961, IFI = 0.971, RMSEA = 0.058 < 0.08.

Flow experience (FE): FE measures were adopted from Novak et al.’s 3-item scale [123]. For example, “My attention is completely focused on what I am doing” and “I feel a sense of control and mastery over the platform work”. The scale has a single dimension, and Cronbach’s α value is 0,802. The composition reliability (CR) and the average variance extraction (AVE) all meet the standards, indicating that each variable has good convergence validity.

Burnout: We selected the scale developed by Maslach [124]. The scale has 5 items, such as “I often feel exhausted because of the work of the online platform” and “When I finish the whole day’s delivery or driving, I feel very tired”. The scale has a single dimension, and Cronbach’s α value is 0,867. A confirmatory factor analysis (CFA) was performed and indicated that the scale demonstrated a satisfactory fit with the data: χ2/df = 2.505 < 3, GFI = 0.987, NFI = 985, CFI = 0.991, TLI = 0.981, IFI = 0.991, RMSEA = 0.064 < 0.08.

Employee work engagement (EWE): The variable of EWE is measured using the scale developed by Schaufeli et al. [3], with a total of 16 items. such as “I’m proud of my work” and “Once I get up in the morning, I want to work on the platform as soon as possible”. The scale has three dimensions, and Cronbach’s α values are 0.884, 0.876 and 0.905 respectively. A confirmatory factor analysis (CFA) was performed and indicated that the scale demonstrated a satisfactory fit with the data: χ2/df = 1.487 < 3, GFI = 0.951, NFI = 957, CFI = 0.985, TLI = 0.982, IFI = 0.985, RMSEA = 0.037 < 0.08.

Control variables: Previous studies have shown that gig workers’ age, gender, education, and type of employment (full or part-time) tenure have a certain impact on gig workers’ work engagement. Therefore, we controlled for the impact of these four variables.

## Results

### Sample characteristics

A total of 400 questionnaires were collected this time. After deleting the items with incomplete filling and filling time less than 1 min, 365 valid questionnaires were obtained, and the overall response rate of questionnaire recovery was 91.25%. Among the valid samples, in terms of gender, men accounted for 64.7% and women accounted for 35.3%; in terms of age, 16.7% were less than 20 years old, 57.3% were between 20 and 30 years old, and 26% were 30 years old and above; in terms of education, 26.3% and below, 59.7% of junior college or undergraduate, 14% of master’s degree and above; in terms of occupation type, 76.7% of full-time, part-time 23.3%.

### Common method deviation test

In the questionnaire design and collection process, this paper has controlled the deviation of common methods by expanding sample sources, anonymity, and rewriting back translation. In this paper, Harman single factor test is used to test [125], exploratory factor analysis is carried out on all variables, and the results of unrotated factor analysis are tested. If only one factor is separated or one factor has particularly strong explanatory power, it is judged that there is a serious common method deviation. Eight factors were separated by exploratory factor analysis, and the variation explained by the factor with the largest variance was 31.165%, which was less than 40%, indicating no serious common method deviation.

### Reliability and validity test

In this paper, confirmatory factor analysis is used to test the rationality of the measurement model, including the reliability, aggregation validity, and discrimination validity of each variable. The results of reliability and aggregate validity are listed in Table 1. Specifically, the results show that Cronbach’s alpha values of all variables exceed 0.70, and the CR of all variables is greater than 0.70, which meets the general requirements of reliability evaluation. The polymerization effect was measured by factor load coefficient and AVE. The results show that the factor loads of all variables are between 0.70 and 0.87, all exceeding 0.70, and the AVE of all variables is between 0.56 and 0.58, all exceeding 0.50. The above results show that the measurement model has good reliability and internal consistency reliability.

**Table 1:** Value of CR and AVE (N = 365)

Variables	Items	Factor Loading	C.R.	P	Cronbach′s α	CR	AVE
PAC	*guide*	*0.77*			*0.88*	*0.79*	*0.56*
	*assess*	*0.70*	*7.44*	***			
	*restrain*	*0.81*	*7.05*	***			
Burnout	*Buruout1*	*0.78*			*0.86*	*0.86*	*056*
	*Buruout2*	*0.78*	*14.99*	***			
	*Buruout3*	*0.71*	*13.60*	***			
	*Buruout4*	*0.70*	*13.45*	***			
	*Buruout5*	*0.78*	*15.04*	***			
FE	*FE1*	*0.71*			*0.80*	*0.80*	*0.58*
	*FE2*	*0.78*	*11.64*	***			
	*FE3*	*0.80*	*11.62*	***			
EWE	*vigor*	*0.73*			*0.91*	*0.80*	*0.57*
	*contribution*	*0.71*	*8.44*	***			
	*concentration*	*0.87*	*8.42*	***			

Notes: *** *p* < 0.001; N = 365

The discrimination validity test results of the measurement model are shown in Table 2. As can be seen from Table 2, the square root of the AVE value of each variable (the bold number in the diagonal in the table) is greater than the correlation coefficient between the variables, which shows that the discrimination validity of the measurement model meets the modelling requirements. From the above analysis, the measurement model in this paper has good reliability and validity and meets the conditions of structural modelling measurement and testing.

**Table 2:** The correlation coefficient and discriminant validity between variables (N = 365)

Variables	*PAC*	*Burnout*	*FE*	*EWE*
PAC	***0.74***			
Burnout	0.43**	***0.75***		
FE	0.32**	0.16**	***0.76***	
EWE	0.42**	0.64**	0.28**	***0.76***

Notes: ** *p* < 0.01 The diagonal black bold value is the square root of AVE.

### Test of the structural model

**Fig. 2:**
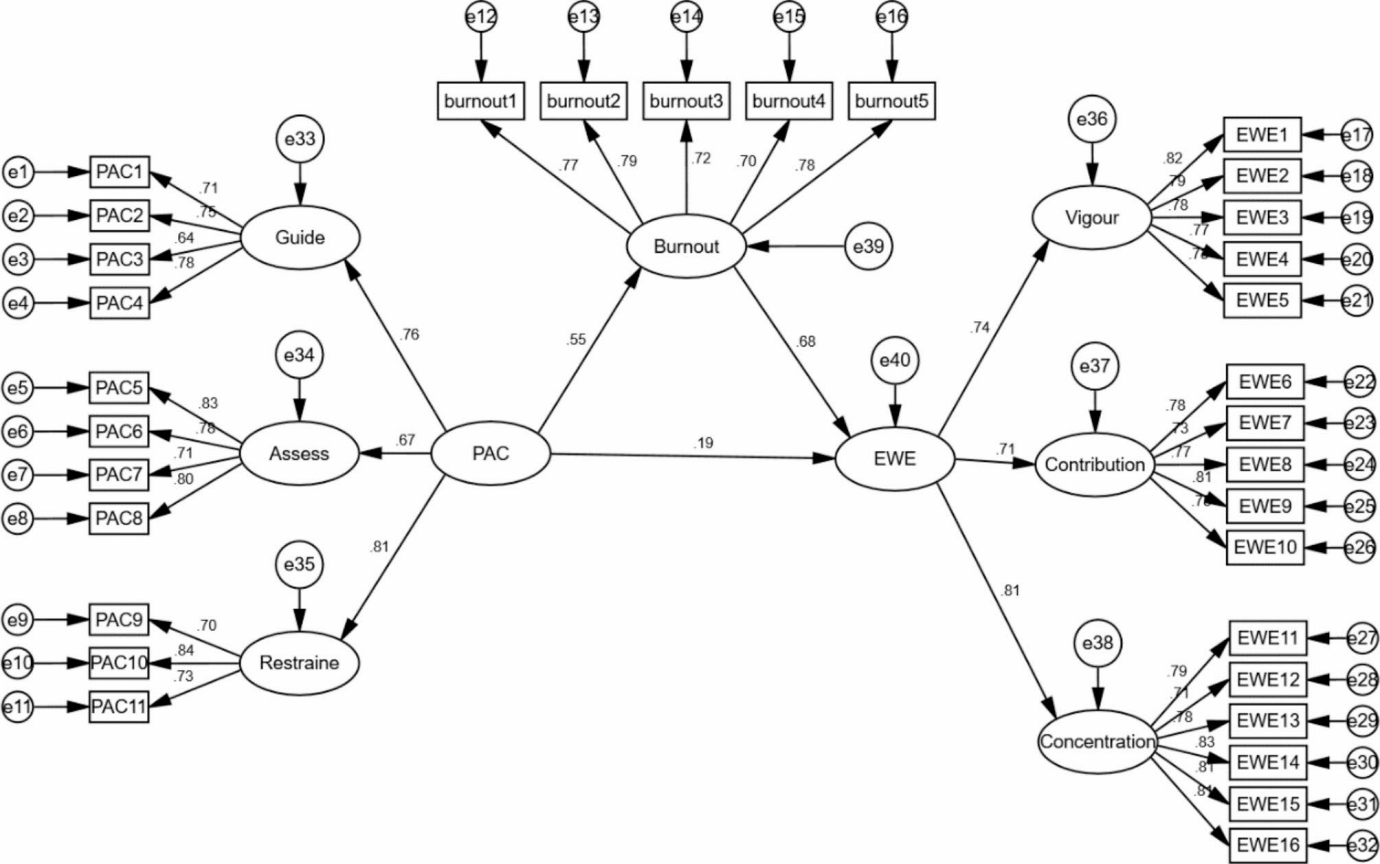
Structural equation model path

In this paper, according to the research theory and research hypothesis, the influence relationship model between variables is constructed, and the influence structure equation path between variables is shown in Fig. 2.

This study uses AMOS 23.0 software for structural equation analysis, and the specific fitting index is shown in Table 3.

**Table 3:** Test results of model fitting degree

index	Standard value	statistical values	conclusion
*CMIN/DF*	*< 3.0*	*1.35*	*fine*
*RMSEA*	*< 0.08*	*0.03*	*fine*
*RMR*	*< 0.08*	*0.06*	*fine*
*GFI*	*> 0.80*	*0.90*	*fine*
*CFI*	*> 0.90*	*0.97*	*fine*
*AGFI*	*> 0.80*	*0.89*	*acceptable*
*NFI*	*> 0.90*	*0.90*	*fine*
*IFI*	*> 0.90*	*0.97*	*fine*
*TLI*	*> 0.90*	*0.97*	*fine*

As can be seen from the above table, the CMIN/DF is 1.35, which is significantly less than 3.0, and other indicators also perform well, indicating that the overall fitting of the model is good. The hypothetical theoretical model in this paper is in good agreement with the actual data, and the model is convincing.

#### The structural model test

The final structural model test results are shown in Table 4. As can be seen, PAC has a significant positive effect on burnout (β = 0.55, p < 0.05), and hypothesis 2 is supported. That is to say, the higher the perceived algorithmic control, the more obvious burnout. PAC has a significant positive effect on EWE (β = 0.18, p < 0.05), and hypothesis 1 is supported. Burnout has a significant positive effect on EWE (β = 0.67, p < 0.05), and hypothesis 3 is supported. Although the sense of burnout is obvious, gig workers will still show high work engagement.

**Table 4:** Basic path coefficient of the model

path			Standardization coefficient	Non-standardized coefficient	S.E.	C.R.	P	hypothesis
*Burnout*	←	*PAC*	*0.55*	*0.67*	*0.09*	*6.94*	***	*hold*
*EWE*	←	*PAC*	*0.18*	*0.20*	*0.07*	*2.62*	*0.00*	*hold*
*EWE*	←	*Burnout*	*0.67*	*0.59*	*0.07*	*8.21*	***	*hold*

Notes: *** *p* < 0.001

#### The mediating effect test

From Table 5, the total effect value of PAC on EWE is 0.56, which does not include 0 within the Lower and Upper values of Bias-Corrected and Percentile 95% CI, indicating that the total effect exists. The indirect effect of PAC on EWE through Burnout is 0.37, which does not include 0 in the range of Lower and Upper of Bias-Corrected and Percentile 95% CI, indicating the existence of an indirect effect; The value of PAC’s direct effect on EWE is 0.18, which does not include 0 in the range of Lower and Upper values of Bias-Corrected and Percentile 95% CI, indicating the existence of the direct effect. Therefore, burnout partially mediates the relationship between PAC and EWE, and Hypothesis 4 is supported.

**Table 5:** Mediating Role of Burnout

Variables	Standardized effect value	Bias-Corrected		Percentile	
		95% CI		95% CI	
		Lower	Upper	Lower	Upper
***Total effect***					
PAC-EWE	*0.56*	*0.39*	*0.73*	*0.38*	*0.73*
***Indirect effect***					
PAC-Burnout-EWE	*0.37*	*0.29*	*0.47*	*0.28*	*0.46*
***Direct effect***					
PAC-EWE	*0.18*	*0.05*	*0.34*	*0.04*	*0.34*

#### The moderating effect of FE

It can be seen from Table 6 that Burnout has a positive effect on EWE (β = 0.64, p < 0.001, model 2), and the interaction coefficient between burnout and FE(Burnout x FE) has a significant positive effect on EWE (β = 0.11, p < 0.05), which indicates that FE plays a moderating role in the relationship between burnout and EWE. Thus, Hypothesis 5 is supported.

**Table 6:** Moderating Role of FE

Variables	EWE			
	Model 1	Model 2	Model 3	Model 4
	β	β	β	β
Gender	*0.07*	*0.03*	*0.03*	*0.03*
Age	*0.07*	*0.07*	*0.05*	*0.06*
Education	0.11*	*0.04*	*0.04*	*0.04*
Type of employment	*0.05*	*0.00*	*0.00*	*0.00*
Burnout		0.64***	0.61***	0.62***
FE			0.18***	0.18***
Burnout x FE				0.11**
R2	*0.02*	*0.43*	*0.46*	*0.47*
ΔR2	*0.02*	*0.40*	*0.03*	*0.01*
F	2.50*	53.84***	50.67***	45.50***

Notes: * *p* < 0.05. ** *p* < 0.01. *** *p* < 0.001; N = 365

**Fig. 3:**
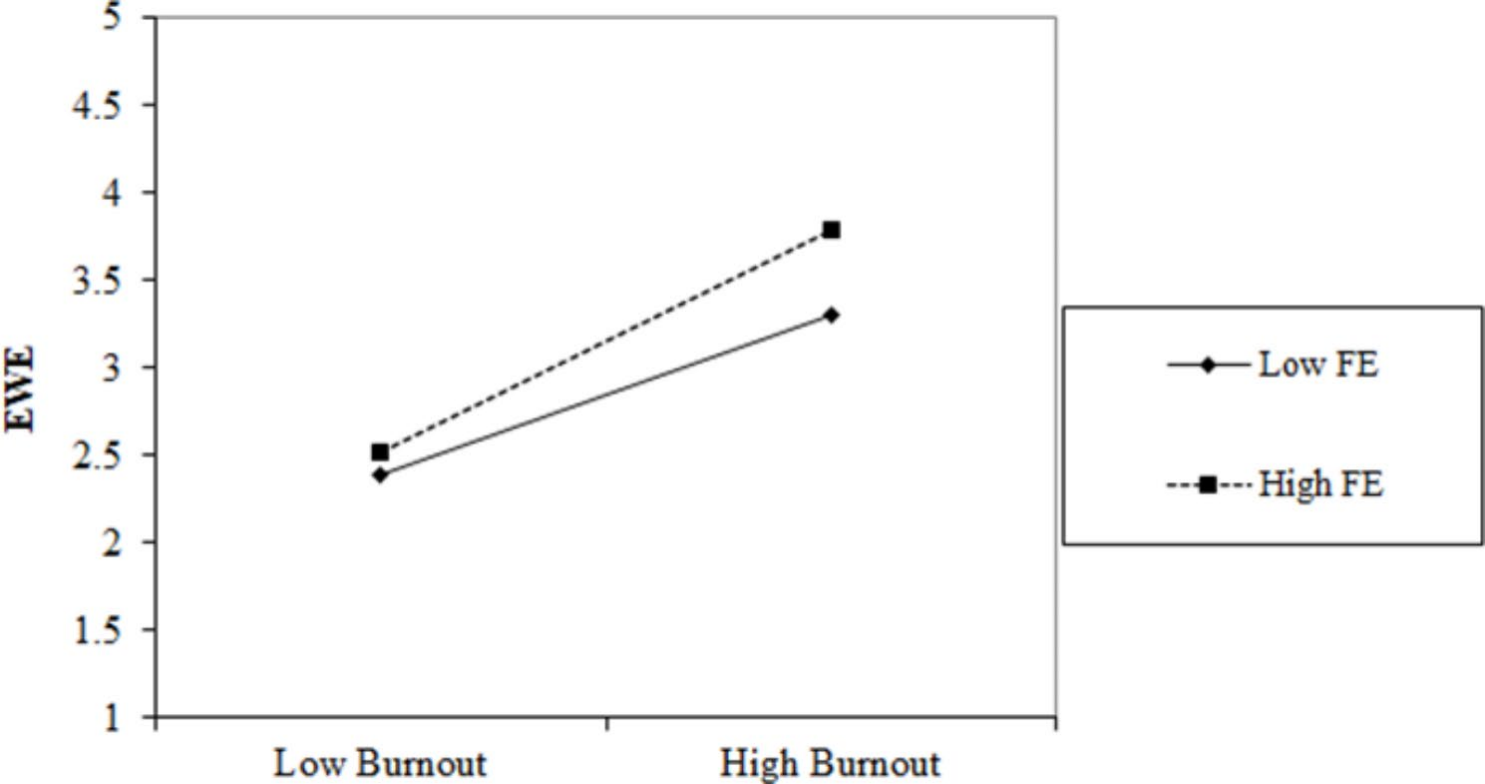
The Moderating Effect of FE between Burnout and EWE

To present the moderating effect of FE more vividly, this study drew the moderating effect diagram of high FE and low FE (see Fig. 3). As seen in Fig. 3, compared with employees with low FE, employees with high FE can strengthen the positive impact of burnout on EWE. In other words, when employees have a high FE, gig workers who feel burnout will also maintain a high level of work engagement. Thus, hypothesis 5 is again supported.

#### The moderated mediating effect test

This study uses the SPSS Process software plug-in, selects model 14, and obtains the results of the moderated mediation effect of FE based on 5000 Bootstrap sampling tests. As Table 7 shows, under low FE, the mediating effect is 0.22 (confidence interval [0.14, 0.22]); under high FE, the mediating effect is 0.31(confidence interval [0.29, 0.40]). Confidence intervals do not contain 0, and the moderated mediating effect is significant. In addition, the mediation effect of FE low is less than FE High, the corresponding moderated mediation test value is 0.04, and the corresponding confidence interval does not contain 0. Again, it is verified that the moderated mediating effect is significant. Thus, Hypothesis 6 is supported.

**Table 7:** The moderated mediating effect test

	Mediator					INDEX of MODERATED MEDIATION			
	Moderator	Effect	BootSE	BootLLCI	BootULCI	Index	SE(Boot)	BootLLCI	BootULCI
Burnout	*FE low*	*0.22*	*0.04*	*0.14*	*0.29*	*0.04*	*0.02*	*0.00*	*0.10*
	*FE High*	*0.31*	*0.04*	*0.22*	*0.40*				

## Discussion

The digital economy, driven by big data technology, breaks through traditional transaction logic and organizational management methods, such that the supply and demand sides are connected through the platform, to achieve value co-creation [126]. The emergence of the gig economy has promoted a flexible and digital employment form. With the continuous development of gig economy, why do some people finally choose gig jobs while others do not [127, 128]? The influence of algorithmic control on the emotional experience and work engagement of gig workers deserves attention and research. In this context, this study analyzed the influence of algorithmic control on the psychological state of gig workers and the mediating effect of psychological changes on work engagement.

Firstly, hypothesis 1 is verified, that is, gig workers’ perceptions of algorithmic control positively affect employees’ work engagement. The results of this study are consistent with that of Moeller (2018) [47]. Employee work engagement is mainly taken as a variable in corporate ethics and organizational behavior. Our findings complement and validate the organizational climate and cognitive experiences that influence employee work engagement by responding to Muller’s call to apply these theories to a digitally mediated platform [129], demonstrating that many of the claims made in the context of traditional organizations may also hold in the digital environment. In this sense, this study is both background and empirical. This paper extends the field of research on employee work engagement and bridges the gap between perceived algorithmic control and employee work engagement by expanding upon research that examines the interplay between employee work engagement and the adoption of digital information technology. Based on the background of digital transformation, the influence of the change in organizational environment and the flattening of organizational structure on employees’ job engagement has aroused discussion. The conclusion of this paper supplements and verifies the organizational climate and emotional perception of gig workers and provides a new idea for the development of organizational behavior and the optimization of organizational environment under the algorithm technology.

Secondly, hypothesis 2 and hypothesis 3 are supported, that is, burnout played a partial mediating role in the relationship between perceived algorithmic control and employee work engagement. It is proved in this paper that perceived algorithmic control will cause negative emotions in gig workers. Although the intelligent matching and control of big data managed by algorithms can improve the management cost and performance of enterprises, the diversity of values and preferences are ignored in the process of algorithm encouragement, which makes the decision-making of rewards and punishments slightly “automated” and “dehumanized“ [86], which makes gig workers face both physical and emotional pressures. Besides, this paper finds an interesting conclusion: burnout will positively affect employee work engagement. Although gig workers are tired of algorithmic control, they will still maintain a high degree of work engagement. For one thing, in the face of an increasingly competitive society, employees’ adaptive behavior is normal in organizational life [103]. At the same time, based on the JD-R model, algorithm technology and big data provide the necessary work resources for gig workers, and the incentive of work resources promotes employees’ work engagement.

Thirdly, the moderating effect of flow experience on the positive relationship between perceived algorithmic control and burnout was verified. Flow experience reflects the immersive experience of employees in the working state, and it is also an important antecedent variable of employee work engagement [130]. As far as organizational management practice is concerned, business operators certainly hope that the organization and employees can achieve an all-round fit. This study discusses the significance of flow experience in weakening burnout and further promoting employees’ work engagement under the background of gig economy, which enriches the application scope of flow experience in a new organizational environment. In addition, this paper combined the JD-R model and added the variable of flow experience, which provided a novel explanation framework for algorithmic control of the theoretical model that affected employees’ job engagement.

### Practical inspiration

Although data-driven programs replace human managers to a greater extent, algorithmic technology will match gig workers with customers, allocate work, monitor labor process, evaluate performance, and make a series of human resources decisions, which will inevitably weaken the role of traditional human resources departments. However, human development is social communication based on emotion. When human supervisor is replaced by digital system, it may have a long-term negative impact on many aspects. To explore the platform ecology with a positive data value cycle, platform enterprises should pay attention to the role of Internet technology and platform operation mechanism, emotional labor, time embedding and other factors in algorithm management. Specifically, Platform enterprises should optimize the management of the platform from the perspective of employees, combined with employees’ cognition and feelings, avoid long-term high-intensity algorithm control, and shorten online work time. At the same time, enterprises should set up a certain fault-tolerant mechanism, such as customer praise can offset short-term over time [131], so that gig workers can feel that the algorithm is not only intelligent but also “warm”, thus alleviating their stress, anxiety, and burnout, and further improving their employees’ work engagement. this research shows that an excellent organizational climate (equality, autonomy, fairness, recognition, etc.) can stimulate employee engagement [132]. (2) Dig into the value needs of gig workers, design and develop gamification task allocation mode and humanized operating system through the algorithm and enhance the work pleasure and sense of accomplishment in the digital gig labor process. Based on the actual situation of the enterprise, platform enterprises should take the JD-R model as an important tool for enterprise human resource management, understand the work demand of gig workers clearly and give full play to technical advantages to accurately match work resources. Human resources departments should also participate more in organizational management and information system design, reduce the “algorithm hegemony” brought by algorithms, increase algorithm negotiation and democratic participation procedures oriented to information fairness, procedural fairness, and distribution fairness, improve the atmosphere of working environment, and alleviate the influence of algorithm control on the organizational behavior of gig jobs. The organizational control of gig workers should not be completely handed over to technology, and the combination of technical management and humanistic care can better stimulate the incentive and promotion of flow experience for work engagement. (3) New employees pay more attention to the experience of working and their continuous self-development [133], and a harmonious and orderly organizational climate always goes hand in hand with the corporate cultural environment and employees’ psychological health [134]. Therefore, in the long run, in addition to giving more autonomy to gig workers by flexibly designing the workflow and optimizing the technical elements of the platform, platform enterprises should focus on how to alleviate the dual physical and psychological pressure that gig workers are subjected to because of the algorithmic control of online labor platforms. Furthermore, platform enterprises should encourage a people-oriented corporate culture and aim to achieve a win-win situation about the occupational health of employees and the enterprises’ corporate interests by adopting a perspective that considers employees’ physical and mental health.

### Research limitations and prospects

This study still has some limitations that need to be further promoted in the follow-up research. First, the cross-sectional research design used in this study makes it difficult to collect cross-sectional data to examine the dynamic relationship between the four variables of gig workers’ perceived algorithmic control, burnout, flow experience, and employee work engagement. Future research can collect data at multiple time points to thoroughly test the relationship between the above variables and the directionality of the relationship. Second, single-source data leads to common method bias. Future research can increase and adjust the research method, using interview methods rooted in coding, behavioral experiments, and other multiple sources to explore their emotional changes and make the findings more practically meaningful. Third, regional differences may affect the generalizability of the results. The data for this study comes from gig workers in several cities and may not be entirely consistent with other regions. In the future, employee data from several provinces could be collected and analyzed.

The important conclusion of this paper is that although perceived algorithmic control causes employees’ burnout, it positively impacts their work engagement. This conclusion may be explained by introducing human-environment fit theory, algorithmic control, technical support of big data and the work demand of gig workers, which realize the mutual matching between demand and supply, thus having a positive impact on employees’ work engagement. Future research can be further explored in this direction.

## Data Availability

The datasets used and analyzed during the current study are available from the corresponding author upon reasonable request.
